# Supplementary material for: Iterative improvement in the automatic modular design of robot swarms
Source: PeerJ Comput Sci. 2020 Dec 7;6:e322. doi: 10.7717/peerj-cs.322 (PMC7924708; doi:10.7717/peerj-cs.322)
Supplement: Supplemental Information 3 [file peerj-cs-06-322-s003.zip › argos3/doc/api/standalone/a00424_source.html]

ARGoS: plugins/robots/eye-bot/simulator/eyebot\_entity.cpp Source File


- Main Page
- Related Pages
- Namespaces
- Classes
- Files

- File List
- File Members

# plugins/robots/eye-bot/simulator/eyebot\_entity.cpp

Go to the documentation of this file.

```
00001 
00007 #include "eyebot_entity.h"
00008 
00009 #include <argos3/core/simulator/space/space.h>
00010 #include <argos3/core/simulator/entity/controllable_entity.h>
00011 #include <argos3/core/simulator/entity/embodied_entity.h>
00012 #include <argos3/plugins/simulator/entities/led_equipped_entity.h>
00013 #include <argos3/plugins/simulator/entities/light_sensor_equipped_entity.h>
00014 #include <argos3/plugins/simulator/entities/perspective_camera_equipped_entity.h>
00015 #include <argos3/plugins/simulator/entities/proximity_sensor_equipped_entity.h>
00016 #include <argos3/plugins/simulator/entities/quadrotor_entity.h>
00017 #include <argos3/plugins/simulator/entities/rab_equipped_entity.h>
00018 
00019 namespace argos {
00020 
00021    /****************************************/
00022    /****************************************/
00023 
00024    static const Real BODY_HEIGHT              = 0.566f;
00025    static const Real BODY_RADIUS              = 0.25f;
00026    static const Real LED_RING_RADIUS          = BODY_RADIUS + 0.005;
00027    static const Real LED_LOWER_RING_ELEVATION = 0.1535f;
00028    static const Real LED_UPPER_RING_ELEVATION = 0.1635f;
00029    static const CRadians LED_ANGLE_SLICE      = CRadians(3.14159265358979323846264338327950288 / 8.0);
00030    static const CRadians HALF_LED_ANGLE_SLICE = LED_ANGLE_SLICE * 0.5f;
00031    static const Real PROXIMITY_SENSOR_RING_ELEVATION       = LED_UPPER_RING_ELEVATION;
00032    static const Real PROXIMITY_SENSOR_RING_RADIUS          = LED_RING_RADIUS;
00033    static const CRadians PROXIMITY_SENSOR_RING_START_ANGLE = CRadians((ARGOS_PI / 12.0f) * 0.5f);
00034    static const Real PROXIMITY_SENSOR_RING_RANGE           = 3.0f;
00035 
00036    /****************************************/
00037    /****************************************/
00038 
00039    CEyeBotEntity::CEyeBotEntity() :
00040       CComposableEntity(NULL),
00041       m_pcControllableEntity(NULL),
00042       m_pcEmbodiedEntity(NULL),
00043       m_pcLEDEquippedEntity(NULL),
00044       m_pcLightSensorEquippedEntity(NULL),
00045       m_pcPerspectiveCameraEquippedEntity(NULL),
00046       m_pcProximitySensorEquippedEntity(NULL),
00047       m_pcQuadRotorEntity(NULL),
00048       m_pcRABEquippedEntity(NULL) {
00049    }
00050 
00051    /****************************************/
00052    /****************************************/
00053 
00054    CEyeBotEntity::CEyeBotEntity(const std::string& str_id,
00055                                 const std::string& str_controller_id,
00056                                 const CVector3& c_position,
00057                                 const CQuaternion& c_orientation,
00058                                 Real f_rab_range,
00059                                 size_t un_rab_data_size,
00060                                 const CRadians& c_perspcam_aperture,
00061                                 Real f_perspcam_focal_length,
00062                                 Real f_perspcam_range) :
00063       CComposableEntity(NULL, str_id),
00064       m_pcControllableEntity(NULL),
00065       m_pcEmbodiedEntity(NULL),
00066       m_pcLEDEquippedEntity(NULL),
00067       m_pcLightSensorEquippedEntity(NULL),
00068       m_pcPerspectiveCameraEquippedEntity(NULL),
00069       m_pcProximitySensorEquippedEntity(NULL),
00070       m_pcQuadRotorEntity(NULL),
00071       m_pcRABEquippedEntity(NULL) {
00072       try {
00073          /*
00074           * Create and init components
00075           */
00076          /*
00077           * Embodied entity
00078           * Better to put this first, because many other entities need this one
00079           */
00080          m_pcEmbodiedEntity = new CEmbodiedEntity(this, "body_0", c_position, c_orientation);
00081          AddComponent(*m_pcEmbodiedEntity);
00082          /* Quadrotor entity */
00083          m_pcQuadRotorEntity = new CQuadRotorEntity(this, "quadrotor_0");
00084          AddComponent(*m_pcQuadRotorEntity);
00085          /* LED equipped entity, with LEDs [0-11] and beacon [12] */
00086          m_pcLEDEquippedEntity = new CLEDEquippedEntity(this, "leds_0");
00087          m_pcLEDEquippedEntity->AddLEDRing(
00088             CVector3(0.0f, 0.0f, LED_LOWER_RING_ELEVATION),
00089             LED_RING_RADIUS,
00090             HALF_LED_ANGLE_SLICE,
00091             16,
00092             m_pcEmbodiedEntity->GetOriginAnchor());
00093          m_pcLEDEquippedEntity->AddLEDRing(
00094             CVector3(0.0f, 0.0f, LED_UPPER_RING_ELEVATION),
00095             LED_RING_RADIUS,
00096             HALF_LED_ANGLE_SLICE,
00097             16,
00098             m_pcEmbodiedEntity->GetOriginAnchor());
00099          CVector3 cLEDPos(LED_RING_RADIUS * 0.7f,
00100                           0.0f,
00101                           LED_LOWER_RING_ELEVATION - 0.01f);
00102          cLEDPos.RotateZ(3.0f * CRadians::PI_OVER_FOUR);
00103          m_pcLEDEquippedEntity->AddLED(
00104             cLEDPos,
00105             m_pcEmbodiedEntity->GetOriginAnchor());
00106          AddComponent(*m_pcLEDEquippedEntity);
00107          /* Proximity sensor equipped entity */
00108          m_pcProximitySensorEquippedEntity =
00109             new CProximitySensorEquippedEntity(this,
00110                                                "proximity_0");
00111          AddComponent(*m_pcProximitySensorEquippedEntity);
00112          m_pcProximitySensorEquippedEntity->AddSensorRing(
00113             CVector3(0.0f, 0.0f, PROXIMITY_SENSOR_RING_ELEVATION),
00114             PROXIMITY_SENSOR_RING_RADIUS,
00115             PROXIMITY_SENSOR_RING_START_ANGLE,
00116             PROXIMITY_SENSOR_RING_RANGE,
00117             24,
00118             m_pcEmbodiedEntity->GetOriginAnchor());
00119          /* Light sensor equipped entity */
00120          m_pcLightSensorEquippedEntity =
00121             new CLightSensorEquippedEntity(this,
00122                                            "light_0");
00123          AddComponent(*m_pcLightSensorEquippedEntity);
00124          m_pcLightSensorEquippedEntity->AddSensorRing(
00125             CVector3(0.0f, 0.0f, PROXIMITY_SENSOR_RING_ELEVATION),
00126             PROXIMITY_SENSOR_RING_RADIUS,
00127             PROXIMITY_SENSOR_RING_START_ANGLE,
00128             PROXIMITY_SENSOR_RING_RANGE,
00129             24,
00130             m_pcEmbodiedEntity->GetOriginAnchor());
00131          /* RAB equipped entity */
00132          m_pcRABEquippedEntity = new CRABEquippedEntity(
00133             this,
00134             "rab_0",
00135             un_rab_data_size,
00136             f_rab_range,
00137             m_pcEmbodiedEntity->GetOriginAnchor(),
00138             *m_pcEmbodiedEntity);
00139          AddComponent(*m_pcRABEquippedEntity);
00140          /* Perspective camera equipped entity */
00141          CQuaternion cPerspCamOrient(CRadians::PI_OVER_TWO, CVector3::Y);
00142          SAnchor& cPerspCamAnchor = m_pcEmbodiedEntity->AddAnchor("perspective_camera",
00143                                                                   CVector3(0.0, 0.0, 0.0),
00144                                                                   cPerspCamOrient);
00145          m_pcPerspectiveCameraEquippedEntity =
00146             new CPerspectiveCameraEquippedEntity(this,
00147                                                  "perspective_camera_0",
00148                                                  c_perspcam_aperture,
00149                                                  f_perspcam_focal_length,
00150                                                  f_perspcam_range,
00151                                                  640, 480,
00152                                                  cPerspCamAnchor);
00153          AddComponent(*m_pcPerspectiveCameraEquippedEntity);
00154          /* Controllable entity
00155             It must be the last one, for actuators/sensors to link to composing entities correctly */
00156          m_pcControllableEntity = new CControllableEntity(this, "controller_0");
00157          AddComponent(*m_pcControllableEntity);
00158          m_pcControllableEntity->SetController(str_controller_id);
00159          /* Update components */
00160          UpdateComponents();
00161       }
00162       catch(CARGoSException& ex) {
00163          THROW_ARGOSEXCEPTION_NESTED("Failed to initialize entity \"" << GetId() << "\".", ex);
00164       }
00165    }
00166 
00167    /****************************************/
00168    /****************************************/
00169 
00170    void CEyeBotEntity::Init(TConfigurationNode& t_tree) {
00171       try {
00172          /*
00173           * Init parent
00174           */
00175          CComposableEntity::Init(t_tree);
00176          /*
00177           * Create and init components
00178           */
00179          /*
00180           * Embodied entity
00181           * Better to put this first, because many other entities need this one
00182           */
00183          m_pcEmbodiedEntity = new CEmbodiedEntity(this);
00184          AddComponent(*m_pcEmbodiedEntity);
00185          m_pcEmbodiedEntity->Init(GetNode(t_tree, "body"));
00186          /* Quadrotor entity */
00187          m_pcQuadRotorEntity = new CQuadRotorEntity(this, "quadrotor_0");
00188          AddComponent(*m_pcQuadRotorEntity);
00189          /* LED equipped entity, with LEDs [0-11] and beacon [12] */
00190          m_pcLEDEquippedEntity = new CLEDEquippedEntity(this, "leds_0");
00191          m_pcLEDEquippedEntity->AddLEDRing(
00192             CVector3(0.0f, 0.0f, LED_LOWER_RING_ELEVATION),
00193             LED_RING_RADIUS,
00194             HALF_LED_ANGLE_SLICE,
00195             16,
00196             m_pcEmbodiedEntity->GetOriginAnchor());
00197          m_pcLEDEquippedEntity->AddLEDRing(
00198             CVector3(0.0f, 0.0f, LED_UPPER_RING_ELEVATION),
00199             LED_RING_RADIUS,
00200             HALF_LED_ANGLE_SLICE,
00201             16,
00202             m_pcEmbodiedEntity->GetOriginAnchor());
00203          CVector3 cLEDPos(LED_RING_RADIUS * 0.7f,
00204                           0.0f,
00205                           LED_LOWER_RING_ELEVATION - 0.01f);
00206          cLEDPos.RotateZ(3.0f * CRadians::PI_OVER_FOUR);
00207          m_pcLEDEquippedEntity->AddLED(
00208             cLEDPos,
00209             m_pcEmbodiedEntity->GetOriginAnchor());
00210          AddComponent(*m_pcLEDEquippedEntity);
00211          /* Proximity sensor equipped entity */
00212          m_pcProximitySensorEquippedEntity =
00213             new CProximitySensorEquippedEntity(this,
00214                                                "proximity_0");
00215          AddComponent(*m_pcProximitySensorEquippedEntity);
00216          m_pcProximitySensorEquippedEntity->AddSensorRing(
00217             CVector3(0.0f, 0.0f, PROXIMITY_SENSOR_RING_ELEVATION),
00218             PROXIMITY_SENSOR_RING_RADIUS,
00219             PROXIMITY_SENSOR_RING_START_ANGLE,
00220             PROXIMITY_SENSOR_RING_RANGE,
00221             24,
00222             m_pcEmbodiedEntity->GetOriginAnchor());
00223          /* Light sensor equipped entity */
00224          m_pcLightSensorEquippedEntity =
00225             new CLightSensorEquippedEntity(this,
00226                                            "light_0");
00227          AddComponent(*m_pcLightSensorEquippedEntity);
00228          m_pcLightSensorEquippedEntity->AddSensorRing(
00229             CVector3(0.0f, 0.0f, PROXIMITY_SENSOR_RING_ELEVATION),
00230             PROXIMITY_SENSOR_RING_RADIUS,
00231             PROXIMITY_SENSOR_RING_START_ANGLE,
00232             PROXIMITY_SENSOR_RING_RANGE,
00233             24,
00234             m_pcEmbodiedEntity->GetOriginAnchor());
00235          /* RAB equipped entity */
00236          Real fRange = 3.0f;
00237          GetNodeAttributeOrDefault(t_tree, "rab_range", fRange, fRange);
00238          UInt32 unDataSize = 10;
00239          GetNodeAttributeOrDefault(t_tree, "rab_data_size", unDataSize, unDataSize);
00240          m_pcRABEquippedEntity = new CRABEquippedEntity(
00241             this,
00242             "rab_0",
00243             unDataSize,
00244             fRange,
00245             m_pcEmbodiedEntity->GetOriginAnchor(),
00246             *m_pcEmbodiedEntity);
00247          AddComponent(*m_pcRABEquippedEntity);
00248          /* Perspective camera equipped entity */
00249          bool bPerspCamFront = true;
00250          GetNodeAttributeOrDefault(t_tree, "camera_front", bPerspCamFront, bPerspCamFront);
00251          Real fPerspCamFocalLength = 0.035;
00252          GetNodeAttributeOrDefault(t_tree, "camera_focal_length", fPerspCamFocalLength, fPerspCamFocalLength);
00253          Real fPerspCamRange = 2.0;
00254          GetNodeAttributeOrDefault(t_tree, "camera_range", fPerspCamRange, fPerspCamRange);
00255          CDegrees cAperture(30.0f);
00256          GetNodeAttributeOrDefault(t_tree, "camera_aperture", cAperture, cAperture);
00257          CQuaternion cPerspCamOrient(CRadians::PI_OVER_TWO, CVector3::Y);
00258          SAnchor& cPerspCamAnchor = m_pcEmbodiedEntity->AddAnchor("perspective_camera",
00259                                                                   CVector3(0.0, 0.0, 0.0),
00260                                                                   cPerspCamOrient);
00261          m_pcPerspectiveCameraEquippedEntity =
00262             new CPerspectiveCameraEquippedEntity(this,
00263                                                  "perspective_camera_0",
00264                                                  ToRadians(cAperture),
00265                                                  fPerspCamFocalLength,
00266                                                  fPerspCamRange,
00267                                                  640, 480,
00268                                                  cPerspCamAnchor);
00269          AddComponent(*m_pcPerspectiveCameraEquippedEntity);
00270          /* Controllable entity
00271             It must be the last one, for actuators/sensors to link to composing entities correctly */
00272          m_pcControllableEntity = new CControllableEntity(this);
00273          AddComponent(*m_pcControllableEntity);
00274          m_pcControllableEntity->Init(GetNode(t_tree, "controller"));
00275          /* Update components */
00276          UpdateComponents();
00277       }
00278       catch(CARGoSException& ex) {
00279          THROW_ARGOSEXCEPTION_NESTED("Failed to initialize entity \"" << GetId() << "\".", ex);
00280       }
00281    }
00282 
00283    /****************************************/
00284    /****************************************/
00285 
00286    void CEyeBotEntity::Reset() {
00287       /* Reset all components */
00288       CComposableEntity::Reset();
00289       /* Update components */
00290       UpdateComponents();
00291    }
00292 
00293    /****************************************/
00294    /****************************************/
00295 
00296 #define UPDATE(COMPONENT) if(COMPONENT->IsEnabled()) COMPONENT->Update();
00297 
00298    void CEyeBotEntity::UpdateComponents() {
00299       UPDATE(m_pcRABEquippedEntity);
00300       UPDATE(m_pcLEDEquippedEntity);
00301    }
00302 
00303    /****************************************/
00304    /****************************************/
00305 
00306    REGISTER_ENTITY(CEyeBotEntity,
00307                    "eye-bot",
00308                    "Carlo Pinciroli [ilpincy@gmail.com]",
00309                    "1.0",
00310                    "The eye-bot robot, developed in the Swarmanoid project.",
00311                    "The eye-bot is a quad-rotor developed in the Swarmanoid Project. It is a\n"
00312                    "fully autonomous robot with a rich set of sensors and actuators. For more\n"
00313                    "information, refer to the dedicated web page\n"
00314                    "(http://www.swarmanoid.org/swarmanoid_hardware.php).\n\n"
00315                    "REQUIRED XML CONFIGURATION\n\n"
00316                    "  <arena ...>\n"
00317                    "    ...\n"
00318                    "    <eye-bot id=\"eb0\">\n"
00319                    "      <body position=\"0.4,2.3,0.25\" orientation=\"45,0,0\" />\n"
00320                    "      <controller config=\"mycntrl\" />\n"
00321                    "    </eye-bot>\n"
00322                    "    ...\n"
00323                    "  </arena>\n\n"
00324                    "The 'id' attribute is necessary and must be unique among the entities. If two\n"
00325                    "entities share the same id, initialization aborts.\n"
00326                    "The 'body/position' attribute specifies the position of the bottom point of the\n"
00327                    "eye-bot in the arena. When the robot is untranslated and unrotated, the\n"
00328                    "bottom point is in the origin and it is defined as the middle point between\n"
00329                    "the two wheels on the XY plane and the lowest point of the robot on the Z\n"
00330                    "axis, that is the point where the wheels touch the floor. The attribute values\n"
00331                    "are in the X,Y,Z order.\n"
00332                    "The 'body/orientation' attribute specifies the orientation of the eye-bot. All\n"
00333                    "rotations are performed with respect to the bottom point. The order of the\n"
00334                    "angles is Z,Y,X, which means that the first number corresponds to the rotation\n"
00335                    "around the Z axis, the second around Y and the last around X. This reflects\n"
00336                    "the internal convention used in ARGoS, in which rotations are performed in\n"
00337                    "that order. Angles are expressed in degrees. When the robot is unrotated, it\n"
00338                    "is oriented along the X axis.\n"
00339                    "The 'controller/config' attribute is used to assign a controller to the\n"
00340                    "eye-bot. The value of the attribute must be set to the id of a previously\n"
00341                    "defined controller. Controllers are defined in the <controllers> XML subtree.\n\n"
00342                    "OPTIONAL XML CONFIGURATION\n\n"
00343                    "You can set the emission range of the range-and-bearing system. By default, a\n"
00344                    "message sent by an eye-bot can be received up to 3m. By using the 'rab_range'\n"
00345                    "attribute, you can change it to, i.e., 4m as follows:\n\n"
00346                    "  <arena ...>\n"
00347                    "    ...\n"
00348                    "    <eye-bot id=\"eb0\" rab_range=\"4\">\n"
00349                    "      <body position=\"0.4,2.3,0.25\" orientation=\"45,0,0\" />\n"
00350                    "      <controller config=\"mycntrl\" />\n"
00351                    "    </eye-bot>\n"
00352                    "    ...\n"
00353                    "  </arena>\n\n"
00354                    "You can also set the data sent at each time step through the range-and-bearing\n"
00355                    "system. By default, a message sent by a eye-bot is 10 bytes long. By using the\n"
00356                    "'rab_data_size' attribute, you can change it to, i.e., 20 bytes as follows:\n\n"
00357                    "  <arena ...>\n"
00358                    "    ...\n"
00359                    "    <eye-bot id=\"eb0\" rab_data_size=\"20\">\n"
00360                    "      <body position=\"0.4,2.3,0.25\" orientation=\"45,0,0\" />\n"
00361                    "      <controller config=\"mycntrl\" />\n"
00362                    "    </eye-bot>\n"
00363                    "    ...\n"
00364                    "  </arena>\n\n"
00365                    "Finally, you can change the parameters of the camera. You can set its aperture,\n"
00366                    "focal length, and range with the attributes 'camera_aperture',\n"
00367                    "'camera_focal_length', and 'camera_range', respectively. The default values are:\n"
00368                    "30 degrees for aperture, 0.035 for focal length, and 2 meters for range. Check\n"
00369                    "the following example:\n\n"
00370                    "  <arena ...>\n"
00371                    "    ...\n"
00372                    "    <eye-bot id=\"eb0\"\n"
00373                    "             camera_aperture=\"45\"\n"
00374                    "             camera_focal_length=\"0.07\"\n"
00375                    "             camera_range=\"10\">\n"
00376                    "      <body position=\"0.4,2.3,0.25\" orientation=\"45,0,0\" />\n"
00377                    "      <controller config=\"mycntrl\" />\n"
00378                    "    </eye-bot>\n"
00379                    "    ...\n"
00380                    "  </arena>\n\n"
00381                    ,
00382                    "Usable"
00383       );
00384 
00385    /****************************************/
00386    /****************************************/
00387 
00388    REGISTER_STANDARD_SPACE_OPERATIONS_ON_COMPOSABLE(CEyeBotEntity);
00389 
00390    /****************************************/
00391    /****************************************/
00392 
00393 }
```

---

Generated on 10 Jul 2018 for ARGoS by 
 1.6.1
